# Accelerating Testosterone Prescribing for U.S. Women: Implications for Cardiovascular Safety

**DOI:** 10.1016/j.jacadv.2026.102724

**Published:** 2026-05-06

**Authors:** Ido Avivi, Cynthia A. Stuenkel, Revathy Sampath-Kumar, Ori Ben-Yehuda

**Affiliations:** a Division of Cardiovascular Medicine, Department of Medicine, University of California-San Diego, La Jolla, California, USA; b Division of Endocrinology and Metabolism, Department of Medicine, University of California-San Diego, La Jolla, California, USA

**Keywords:** drug safety, electronic health records, testosterone, Women’s health

**What is the clinical question being addressed?**

How has testosterone prescribing for U.S. women changed, and what are the cardiovascular risk implications?

**What is the main finding?**

Testosterone prescribing rates rose 2.6-fold from 2016 to 2025, predominantly among White midlife women, reaching 357 per 100,000, many with prevalent cardiovascular risk factors.

## Background

Reports of testosterone use in women have surged in popular media, with claims of improved libido, energy, and cognitive clarity fueling demand, particularly among midlife women.^1^ However, no testosterone product is U.S. Food and Drug Administration (FDA)-approved for use in women, long-term safety data are lacking, and cardiovascular risk remains uncertain.^2^ BLISS (BioSante LibiGel Safety Study), designed to assess the long-term cardiovascular safety of testosterone gel in women with hypoactive sexual desire disorder (HSDD), was completed but never published after companion efficacy trials failed to show benefit.^3^ Compared with placebo, testosterone therapy in men was associated with greater progression of noncalcified coronary plaque volume on coronary computed tomography angiography.^4^ Despite these concerns, real-world testosterone prescribing in women is poorly characterized. We used a national electronic health record (EHR) network to describe temporal and demographic patterns of testosterone prescribing for U.S. women.

## Methods

Data were extracted from Epic Cosmos, a data set created in collaboration with a community of health systems using Epic EHR, representing more than 300 million patient records from over 1,915 hospitals and 42,600 clinics. The community represents patients from all 50 states and the District of Columbia, Canada, Lebanon, and Saudi Arabia. This analysis was restricted to U.S. patients.

Eligible patients were adult women (age ≥18 years; legal sex recorded as female) with at least two documented encounters. Patients with diagnoses of gender identity disorder (International Classification of Diseases-10th Revision-Clinical Modification F64.∗) or a personal history of sex reassignment (Z87.890) were excluded. Annual counts of women prescribed any testosterone-containing formulation from January 1, 2016, through December 31, 2025, were identified; exposure rates were calculated using platform-reported denominators for the eligible female population. Prescribing rate trends were assessed using piecewise log-linear regression with a data-driven 2021/2022 breakpoint, year as a continuous variable, and population size as a weight. Demographic and clinical characteristics were described using 2025 data, and between-group differences were compared using chi-square tests. This study used deidentified data and was therefore exempt from ethics review and informed consent.

## Results

**Figure 1:**
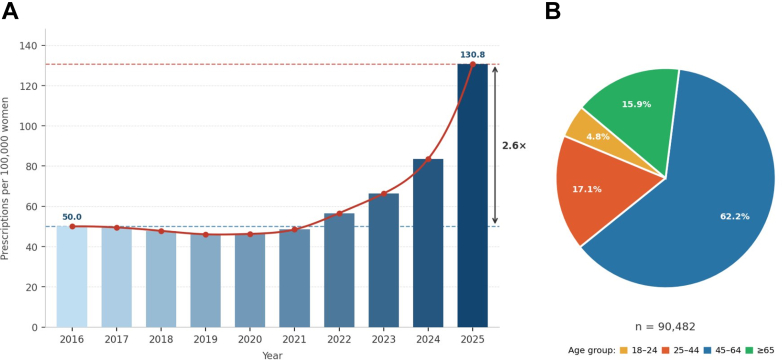
Testosterone Prescribing Trends and Age Distribution for U.S. Women (A) Testosterone prescribing rates remained stable at 46 to 50 per 100,000 from 2016 to 2021, then accelerated to 130.8 per 100,000 in 2025 (2.6-fold increase; *P* < 0.001). (B) Age distribution of women prescribed testosterone in 2025.

Testosterone prescribing rates rose 2.6-fold from 50.0 to 130.8 per 100,000 eligible women between 2016 and 2025 (*P* < 0.001) (Figure 1A), remaining stable through 2021 before increasing at 31.8% per year from 2022 to 2025 (incidence rate ratio: 1.32; 95% CI: 1.25-1.40). This represented 90,482 prescriptions in 2025, a 58.7% year-over-year increase from 2024.

Prescribing was highest for midlife women: in 2025, the 45 to 64 age group accounted for 62.2% of all prescriptions (Figure 1B), with an exposure rate of 274.5 per 100,000—twice the overall population rate—and the greatest year-over-year growth at 79.1% from 2024. The most frequently linked encounter diagnoses were menopausal states (International Classification of Diseases, 10th Revision, Clinical Modification N95.1; 34.6%), decreased libido (R68.82; 24.0%), and hormone replacement therapy (Z79.890; 17.2%). Notably, HSDD (F52.0)—the accepted indication for testosterone prescribing^2^—accounted for only 8.2% of cases. Cardiometabolic risk factors were prevalent: 51.2% had at least 1 documented risk factor (hypertension, dyslipidemia, or type 2 diabetes) and 44.0% had a family history of heart disease; rates similar to the corresponding non-testosterone-prescribed population in Cosmos. However, established ischemic heart disease was less prevalent in the testosterone-prescribed group (3.1% vs 4.7%; *P* < 0.001).

Exposure varied by race and ethnicity. Rates were highest among White (0.17%; 357 per 100,000 in the 45-64 age stratum) and lowest among Black and Asian women (both 0.05%), with intermediate rates among American Indian/Alaska Native (0.12%), Native Hawaiian/Pacific Islander (0.07%), and other race groups (0.15%). White women represented 80.1% of all testosterone recipients. Non-Hispanic women (0.15%) had higher exposure rates than Hispanic/Latino women (0.06%).

## Discussion

In this analysis of a large national EHR network, testosterone prescribing rates for U.S. women increased 2.6-fold from 2016 to 2025, with marked acceleration since 2021. Prescribing was concentrated in midlife women aged 45 to 64; half had documented cardiometabolic risk factors. These findings demonstrate that testosterone prescribing for women is increasingly prevalent—affecting up to 1 in 280—making cardiologists more likely to encounter this exposure and be asked to counsel on cardiovascular safety.

Recorded prescribing-related diagnoses including menopausal symptoms and decreased libido align with claims promoted in popular media.^1^ Per the 2019 Global Consensus Position Statement on the Use of Testosterone in Women, the only evidence-based indication for testosterone is HSDD, as data are insufficient to support use for energy, cognition, or general well-being.^2^ Notably, the Statement recommends against pellets and injections—formulations frequent in media reports^1^—because they result in supraphysiologic testosterone concentrations. No testosterone product is FDA-approved for women, and clinical trials excluded women at high cardiometabolic risk.^2^

Cardiovascular safety data derive predominantly from trials in men. The TRAVERSE (Testosterone Replacement Therapy for Assessment of Long-term Vascular Events and Efficacy Response in Hypogonadal Men) trial demonstrated noninferiority to placebo for major adverse cardiovascular events, prompting FDA replacement of the boxed cardiovascular warning with a blood pressure advisory.^5^ TRAVERSE also showed higher rates of atrial fibrillation (3.5% vs 2.4%; *P* = 0.02) and pulmonary embolism (0.9% vs 0.5). Moreover, the trial only evaluated transdermal testosterone over 22 months, a duration, dose, and formulation that may not reflect real-world use in women. Mechanistic coronary computed tomography angiography studies in men link testosterone therapy to greater noncalcified plaque progression,^4^ potentially portending higher atherosclerotic risk over longer follow-up.

Midlife is a critical period for atherosclerotic disease development in women, with the first myocardial infarction occurring on average 2 decades after menopause. Given sex-specific differences in hormonal milieu, thrombotic susceptibility, treatment indications, and the routes and dosages used in clinical practice,^1^ the increasing prescription of testosterone in midlife women observed in this study warrants dedicated safety assessment. Future research in women should evaluate coronary plaque progression on imaging and real-world comparative safety stratified by formulation, dose, and route of administration.

Our study has limitations. Epic Cosmos may not be nationally representative; demographic, diagnostic, and prescription coding practices and completeness may vary across institutions, potentially affecting subgroup estimates. Prescription data capture intent but not adherence; drug dose, formulation, route of administration, serum testosterone levels, and duration of therapy were not assessed but may differentially affect cardiovascular outcomes. Compounded formulations are likely underascertained. EHR-linked encounter diagnoses may not accurately reflect clinical indications, and a low HSDD coding rate should not be interpreted as evidence of inappropriate prescribing. Although ischemic heart disease prevalence was lower in testosterone-prescribed women, this should not be interpreted as evidence of cardiovascular protection, as confounder adjustment is not available within the Epic Cosmos SlicerDicer platform.

## Conclusions

Testosterone prescribing for U.S. women increased 2.6-fold over the past decade, predominantly in midlife women with prevalent cardiovascular risk factors. Mechanistic trials in men raise concerns about atherosclerotic plaque progression, yet safety data in women remain limited and do not reflect current expanded use.^1^ These findings underscore the need for careful cardiovascular risk assessment and shared decision-making in clinical practice, and highlight the urgency of sex-specific cardiovascular safety studies in this rapidly growing population.

## Funding support and author disclosures

Dr Avivi is supported by the Joanne C. Warren Fellowship Fund. All other authors have reported that they have no relationships relevant to the contents of this paper to disclose.
